# Value of Area Postrema Syndrome in Differentiating Adults With AQP4 vs. MOG Antibodies

**DOI:** 10.3389/fneur.2020.00396

**Published:** 2020-06-04

**Authors:** Jae-Won Hyun, Young Nam Kwon, Sung-Min Kim, Hye Lim Lee, Woo Kyo Jeong, Hye Jung Lee, Byoung Joon Kim, Seung Woo Kim, Ha Young Shin, Hyun-June Shin, Sun-Young Oh, So-Young Huh, Woojun Kim, Min Su Park, Jeeyoung Oh, Hyunmin Jang, Na Young Park, Min Young Lee, Su-Hyun Kim, Ho Jin Kim

**Affiliations:** ^1^Department of Neurology, National Cancer Center, Goyang, South Korea; ^2^Department of Neurology, Seoul National University Hospital, Seoul, South Korea; ^3^The Catholic University of Korea, Eunpyeong St. Mary's Hospital, Seoul, South Korea; ^4^Department of Neurology, Korea University Guro Hospital, Korea University College of Medicine, Seoul, South Korea; ^5^Department of Neurology, Samsung Medical Center, Sungkyunkwan University School of Medicine, Seoul, South Korea; ^6^Department of Neurology, Neuroscience Center, Samsung Medical Center, Seoul, South Korea; ^7^Department of Neurology, Yonsei University College of Medicine, Seoul, South Korea; ^8^Department of Neurology, School of Medicine, Chonbuk National University, Jeonju, South Korea; ^9^Department of Neurology, Kosin University College of Medicine, Busan, South Korea; ^10^Department of Neurology, The Catholic University of Korea, Seoul St. Mary's Hospital, Seoul, South Korea; ^11^Department of Neurology, Yeungnam University College of Medicine, Daegu, South Korea; ^12^Department of Neurology, Konkuk University School of Medicine, Seoul, South Korea

**Keywords:** area postrema syndrome, aquaporin-4 antibody, MOG antibody, neuromyelitis optica spectrum disorder, diagnosis

## Abstract

**Objectives:** To compare the frequency of area postrema syndrome (APS) in adults with anti-aquaporin-4 (AQP4) and anti-myelin oligodendrocyte glycoprotein (MOG) antibodies.

**Methods:** APS is defined as acute or subacute, single or combined, episodic or constant nausea, vomiting, or hiccups, persisting for at least 48 h, which cannot be attributed to any other etiology. The presence of APS was investigated in 274 adults with AQP4 antibodies and 107 adults with MOG antibodies from 10 hospitals.

**Results:** The study population comprised Korean adults (≥18 years). At the time of disease onset, 14.9% (41/274) adults with AQP4 antibodies had APS, while none of the participants with MOG antibodies developed APS (*p* < 0.001). During the course of the disease, 17.2% (47/274) adults with AQP4 antibodies had APS in contrast to 1.9% (2/107) adults with MOG antibodies with APS (*p* < 0.001).

**Conclusions:** APS, one of the core clinical characteristics of individuals with AQP4 antibodies, is an extremely rare manifestation in Korean adults with MOG antibodies.

## Introduction

Individuals with anti-aquaporin-4 (AQP4) antibodies and anti-myelin oligodendrocyte glycoprotein (MOG) antibodies were previously grouped under the umbrella term neuromyelitis optica spectrum disorder (NMOSD), as they shared two cardinal clinical manifestations, optic neuritis and longitudinally extensive transverse myelitis. However, the two conditions are now considered distinct entities based on differences in histopathology, plausible underlying pathogenic mechanisms, clinical courses, treatment responses, and some distinguishing clinical manifestations (1–3).

Area postrema syndrome (APS), one of the core clinical characteristics described in the 2015 diagnostic criteria for NMOSD, is defined as intractable nausea, vomiting, or hiccups, which persist for at least 48 h (4, 5). Area postrema is located in the dorsal tegmentum of the medulla, an AQP4-rich region, which is often affected in individuals with AQP4 antibodies (6–8). In contrast to the preferential expression of AQP4 in certain regions of the central nervous system (CNS) (9), MOG is expressed throughout the CNS. As such, APS is not particularly expected in individuals with MOG antibodies, unlike those with AQP4 antibodies. This study evaluated the value of APS as a clinical characteristic in differentiating individuals with AQP4 antibodies from those with MOG antibodies in a large Korean cohort.

## Methods

The study included 298 participants with AQP4 antibodies from National Cancer Center (NCC) NMOSD cohort and 124 participants with MOG antibodies from 10 referral hospitals between 2005 and 2019. Four non-Koreans and 37 participants (21 with AQP4 antibodies and 16 with MOG antibodies) with the age of onset below 18 years were excluded. Finally, the frequency of APS was evaluated in 274 participants with AQP4 antibodies and 107 participants with MOG antibodies, by retrospective reviewing of the medical records based on physicians' active questioning regarding APS. APS was defined as per the following recently proposed criteria: (1) acute or subacute, single or combined, episodic or constant nausea, vomiting, or hiccups, (2) persistent for at least 48 h, (3) without a known etiology (5). The serostatus of AQP4 and MOG antibodies was assessed using live cell-based assays performed at the NCC and Seoul National University Hospital (10–12).

Fisher's exact test was used to compare the presence of APS between the two groups. The study protocol was approved by the institutional review boards of the NCC.

## Results

### Demographics

The female-to-male ratio was 7.1:1 and 1.1:1 in participants with AQP4 antibodies and MOG antibodies, respectively. All the participants enrolled in this study were Korean. The mean age at disease onset was 37 years (range, 18–80 years) and 36 years (range, 18–72 years) for participants with AQP4 and MOG antibodies, respectively. The mean disease duration was 11 years (range, 1–36 years) and 7 years (range, 1–30 years) in participants with AQP4 and MOG antibodies, respectively. The mean follow-up period was 6 years (range, 1–14 years) and 4 years (range, 1–16 years) in individuals with AQP4 and MOG antibodies, respectively. The mean total number of attacks was 6 (range, 1–36) and 3 (range, 1–12) in participants with AQP4 and MOG antibodies, respectively.

### Presence of APS

**Figure 1:**
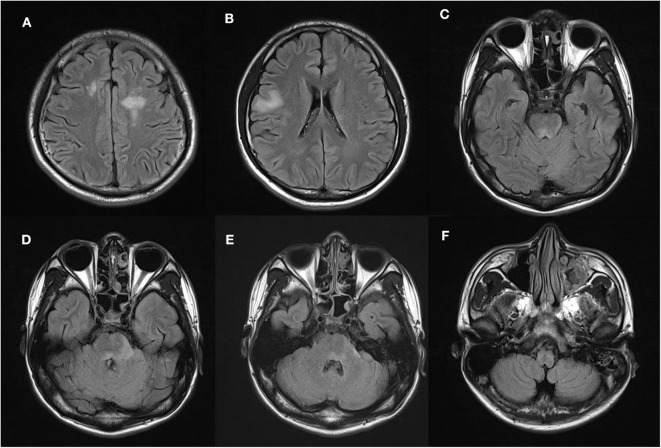
Participant 1 shows high fluid-attenuated inversion recovery (FLAIR) signal abnormalities in **(A,B)** bilateral cerebral hemispheres, **(C)** poorly demarcated dorsal midbrain, and **(D,E)** pontine lesions around the fourth ventricle. **(F)** No area postrema lesion is observed.

**Figure 2:**
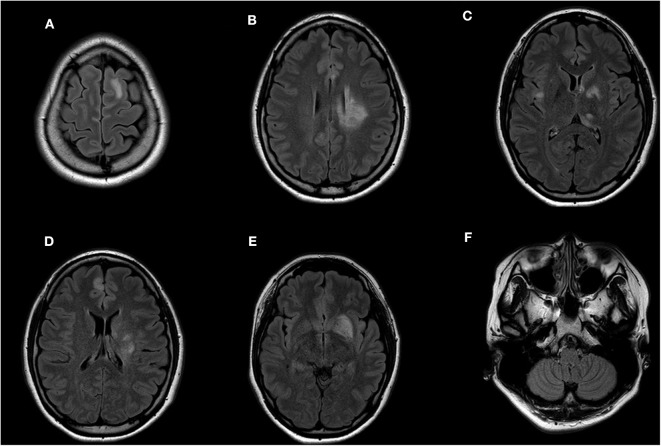
Participant 2 had high FLAIR signal abnormalities in the **(A)** left frontal lobe, **(B–E)** basal ganglia, **(C)** thalamus, right external capsule, and **(D)** right frontal lobe. **(F)** No area postrema lesion is observed.

The initial manifestations of APS were observed in 41 (14.9%) of 274 participants with AQP4 antibodies, while none were observed in participants with MOG antibodies (*p* < 0.001). During the course of the disease, APS occurred in 47 of 274 participants (17.2%) with AQP4 antibodies, while only 2 of 107 participants (1.9%) with MOG antibodies experienced APS (*p* < 0.001). After considering the number of attacks, the significant difference in frequency of APS remained [2.8% (47/1686) vs. 0.6% (2/310), number of APS/total attacks, *p* = 0.026]. One of the two participants with MOG antibodies reported a 1-week history of constant nausea associated with acute disseminated encephalomyelitis (ADEM)-like lesions in the bilateral cerebral hemispheres and poorly demarcated dorsal midbrain and pontine lesions around the fourth ventricle (Figure 1). The other one with MOG antibody had episodic nausea and vomiting for 2 days with ADEM-like patch lesions in the left frontal lobe, basal ganglia, thalamus, and right external capsule (Figure 2). Four other patients with MOG antibodies presented with episodic or constant nausea/vomiting (*n* = 3), or hiccups (*n* = 1), but their symptoms did not persist for at least 48 h. Of six participants with AQP4 antibodies who developed APS after their initial presentation, four showed isolated APS as main phenotype of relapse (two of four with brain MRI at the time of relapse had no or only non-specific brain lesion except area postrema lesion). The remaining two participants with AQP4 antibodies experienced APS with optic neuritis (*n* = 1) or myelitis (*n* = 1) at the time of relapse.

### Discussions

In contrast to AQP4 antibody-positive NMOSD, APS was rarely observed in Korean adults with MOG antibodies: none at onset and only 1.9% during the course of the disease. As the first manifestation, APS was exclusively observed in Korean adults with AQP4 antibodies while two adults with MOG antibodies had APS in the context of ADEM in their subsequent attacks. None of the participants with MOG antibodies had APS with area postrema lesion.

Two previous studies conducted in Western countries reported similar findings in both children and adults; APS at disease onset was rare in children with MOG antibodies (3.8%, 1/26) compared to those with AQP4 antibodies (50%, 4/8) (13), and APS presented only as a subsequent attack (2%, 1/50) in adults with MOG antibodies (14). Most recent article published after the completion of our study reported that the frequency of APS in the context of ADEM was 8.5% (10/117), while APS associated with area postrema lesion was extremely rare (0.9%, 1/117) in adults with MOG antibodies (15). Another recent study focused on magnetic resonance imaging findings also showed that area postrema lesions were uncommon in individuals with MOG antibodies (7%, 1/14) compared to those with AQP4 antibodies (50%, 8/16) (16). However, one study reported a relatively high frequency of APS (14.6%, 11/75) in a Caucasian and adult predominant cohort with MOG antibodies (17). Of note, the duration of patients' symptoms suggestive of APS in this study is uncertain and may have not persisted for at least 48 h, as defined by the criteria (5, 17).

Owing to the retrospective design of the current study based on a cohort of referral hospitals, inevitable potential recall and selection bias in the evaluation of APS might be present; larger prospective population-based studies are warranted to confirm our findings.

In conclusion, APS is a rare clinical feature in Korean adults with MOG antibodies and a reliable core clinical characteristic in those with AQP4 antibodies. Our findings suggest that a comprehensive evaluation of APS could be helpful in distinguishing individuals with AQP4 antibodies from those with MOG antibodies.

## Data Availability Statement

All datasets generated for this study are included in the article/supplementary material.

## Ethics Statement

The studies involving human participants were reviewed and approved by National Cancer Center. The patients/participants provided their written informed consent to participate in this study.

## Author Contributions

J-WH and HK had full access to all of the data in the study and takes responsibility for the integrity of the data and the accuracy of the data analysis, study concept and design and drafting of the manuscript. All authors: acquisition, analysis and interpretation of data, and critical revision of the manuscript for important intellectual content.

## Conflict of Interest

J-WH has received a grant from the National Research Foundation of Korea. S-HK has lectured, consulted, and received honoraria from Bayer Schering Pharma, Biogen, Genzyme, Merck Serono, and UCB and received a grant from the National Research Foundation of Korea. S-MK has lectured, consulted, and received honoraria from Bayer Schering Pharma, Genzyme, Merck Serono, Eisai, and UCB; received a grant from the National Research Foundation of Korea and the Korea Health Industry Development Institue Research; is an associated editor of the Journal of Clinical Neurology; has transferred the technology of flow cytometric AQP4-Ab assay to EONE laboratories. HK has received a grant from the National Research Foundation of Korea; received consultancy/speaker fees from Alexion, Celltrion, Eisai, HanAll BioPharma, Merck Serono, Novartis, Sanofi Genzyme, Teva-Handok, and Viela Bio; serves on a steering committee for MedImmune/Viela Bio; is a co-editor for the Multiple Sclerosis Journal and an associated editor for the Journal of Clinical Neurology. The remaining authors declare that the research was conducted in the absence of any commercial or financial relationships that could be construed as a potential conflict of interest.
